# Cutaneous manifestations of WHIM syndrome

**DOI:** 10.1111/1346-8138.17733

**Published:** 2025-04-09

**Authors:** Rutha Adhanom, Caleb Kim, Jennifer Strong, Sophia Martinez, Heidi H. Kong, Isaac Brownell, Philip M. Murphy, David H. McDermott, Leslie Castelo‐Soccio

**Affiliations:** ^1^ Cutaneous Microbiome and Inflammation Section, Dermatology Branch, National Institute of Arthritis and Musculoskeletal and Skin Diseases National Institutes of Health Bethesda Maryland USA; ^2^ Cutaneous Development and Carcinogenesis Section, Dermatology Branch, National Institute of Arthritis and Musculoskeletal and Skin Diseases National Institutes of Health Bethesda Maryland USA; ^3^ Laboratory of Molecular Immunology National Institute of Allergy and Infectious Diseases Bethesda Maryland USA

**Keywords:** genetic disease, inborn, population characteristics, primary immunodeficiency syndromes, health, skin diseases, infectious

## Abstract

Warts, Hypogammaglobulinemia, Infections, and Myelokathexis (WHIM) syndrome is a rare immunodeficiency caused by gain‐of‐function mutations in the chemokine receptor CXCR4. While human papillomavirus (HPV) skin infection (warts) is the dermatological hallmark of the disease, individuals with WHIM have high rates of other skin manifestations that may aid early diagnosis and management. This study was a retrospective review of medical records from a United States National Institutes of Health natural history cohort of patients with WHIM syndrome seen between 2005 and 2024, including a cross‐sectional analysis of cutaneous manifestations and CXCR4 variants. The cohort compromised 45 patients with genetically confirmed WHIM syndrome, 16 men and 29 women, with a mean age of 33.3 years (range, 0–69 years) and mean age at diagnosis of 20.4 years (range, 0–59 years). The cohort exhibited a range of skin manifestations which included cutaneous infections with HPV in 34 (76%) patients, bacteria in 32 (71%) patients, other viruses in 27 (60%) patients, and fungi in 25 (56%) patients. Inflammatory conditions included six (13%) patients with seborrheic dermatitis, five (11%) with contact dermatitis, four (9%) with psoriasis, three (7%) with nummular eczema, and 13 (29%) with other eczematous dermatitis. Despite the young median age, seven (16%) patients had skin cancer. All seven patients had *CXCR4* truncation mutations, while those with a missense mutation (E343K) generally had fewer skin manifestations. Our study found that WHIM syndrome is associated with diverse infectious, inflammatory, and neoplastic skin conditions beyond HPV skin infection.

## 1 INTRODUCTION

Warts, Hypogammaglobulinemia, Infections, and Myelokathexis (WHIM) syndrome, is an inborn error of immunity caused by pathogenic variants in the G‐protein‐coupled chemokine receptor CXCR4.^1^ Impaired CXCR4 intracellular trafficking leads to prolonged interaction with its ligand, CXCL12, resulting in hyperactive signaling and overmature neutrophils remaining in the bone marrow (myelokathexis).^2^ T‐cell receptor excision circle quantification in newborn screening programs for severe, combined immunodeficiency often reveals low levels in newborns with WHIM, potentially serving as an early indicator;^3^ however, only a few cohorts have been reported.^4^, ^5^, ^6^, ^7^, ^8^

Although human papillomavirus (HPV) infection (warts) is the eponymous dermatological manifestation, other skin conditions may also occur and have been reported sporadically in case reports. This study provides a more comprehensive assessment of skin lesions found in WHIM syndrome by a systematic retrospective analysis of a large, predominantly North American, cohort followed at the National Institutes of Health (NIH). Delineating the range of dermatological presentations may facilitate earlier diagnosis by dermatologists, who are often the early consultants who see these patients.

## 2 METHODS

A retrospective review of medical records from a natural history cohort at the NIH (NCT00128973, McDermott/Murphy) was conducted for 45 patients (16 men and 29 women; mean age 33.3 years [range, 0–69 years]; mean age at diagnosis 20.4 years [range, 0–59 years]; two underwent bone marrow transplant, three died, and 42 were living) diagnosed with pathogenic *CXCR4* variant (Table 1). Among them, 29 had a family history of confirmed or presumptive WHIM syndrome, and 16 individuals were confirmed or presumptively de novo (Table S1). Six were diagnosed in infancy.

**TABLE 1:** CXCR4 variants in NIH WHIM cohort.

CXCR4 mutation variant	Mutation type	Number of patients	Number of families
R334X	Truncation	22	10
E343K	Missense	5	1
S324Vfs*20	Frameshift	3	2
S338X	Truncation	3	2
E343X	Truncation	2	1
K327Rfs*17	Frameshift	2	1
V320Efs*23	Frameshift	2	1
S339Cfs*4	Frameshift	2	1
L329Qfs*13	Frameshift	1	1
G336X	Truncation	1	1
S330Qfs*13	Frameshift	1	1
R332Qfs*22	Frameshift	1	1

*Note*: Table of types of CXCR4 variants seen among the 45 patients with Warts, Hypogammaglobulinemia, Infections, and Myelokathexis (WHIM) syndrome at the National Institutes of Health (NIH), spanning 23 different families. Twelve distinct CXCR4 genetic variants within the NIH cohort with WHIM were noted, including a total of 7 frameshift mutations (S324Vfs*20, L329Qfs*13, K327Rfs*17, S339Cfs*4, V320Efs*23, R332Qfs*22, S330Qfs*13), 4 truncation mutations (R334X, S338X, E343X, G336X), and 1 point mutation (E343K) in the cohort with WHIM. The most common variant seen in WHIM Syndrome is truncation mutation R334\u00D7 observed in 10 families in our cohort with WHIM, two families with frameshift mutation S324Vfs*20, two families with truncation mutation S338\u00D7, and one family each with L329Qfs*13, E343K, E343\u00D7, K327Rfs*17, G336\u00D7, S339Cfs*4, V320Efs*23, S330Qfs*13, and R332Qfs*22 mutations.

## 3 RESULTS

### 3.1 Skin manifestations observed in individuals with WHIM syndrome

**FIGURE 1:**
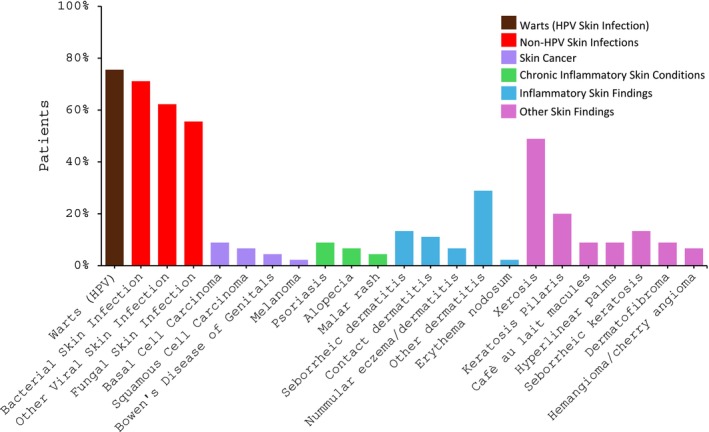
Cutaneous manifestations of Warts, Hypogammaglobulinemia, Infections, and Myelokathexis (WHIM) syndrome. Graphical representation of the cutaneous manifestations observed in 45 patients with WHIM Syndrome at the National Institutes of Health, as detailed in Table S4. The *x*‐axis displays the type of cutaneous manifestations, and the *y*‐axis displays the percentage of patients affected. Each category (skin infection, skin cancer, chronic inflammatory skin conditions, inflammatory skin findings, and other skin findings) is color‐coded according to the legend.

In the cohort, 34 patients (76%) had HPV infection (warts). The wart locations were hands, feet, and anogenital regions for 27 patients (60%), 20 patients (44%), and 14 patients (31%), respectively (Figure 1, Table S2, Figure S1). The distribution of HPV types in this cohort have been previously described^9^, ^10^ and there is an ongoing microbiome study to type new HPV variants. In addition to the more common HPV types (*Alpha* [HPV2, 6, 11]) seen in immunocompetent individuals, WHIM patients have abundant Beta types (HPV5 and 23 predominant) and novel Gamma HPV types. Gamma HPV types have been associated with squamous cell carcinomas.

Non‐HPV skin infections were diagnosed in 40 patients (89%), including 32 patients (71%) with bacterial infection, 27 patients (60%) with non‐HPV viral infection, and 25 patients (56%) with fungal infection (Table 1, Figure S1). Non‐HPV viral skin infections included 11 patients (24%) with herpes simplex virus, nine (20%) with molluscum contagiosum, and eight patients (18%) with varicella zoster virus. Hand, foot, and mouth disease and roseola were seen in two patients (4%) and rubella in one patient (2%; Figure 1, Table S3). Dermatophyte infections were documented 28 times, including eight (18%) cases of tinea corporis, six (13%) of tinea capitis, four (9%) of tinea pedis, and four (9%) cases of onychomycosis. Additional fungal infections included five patients (11%) with candidal diaper rash, three (6%) with other candidal skin rash, six (13%) with tinea versicolor, and one patient (2%) with angular cheilitis (Figure 1). A compilation of all skin findings can be found in Table S4.

**FIGURE 2:**
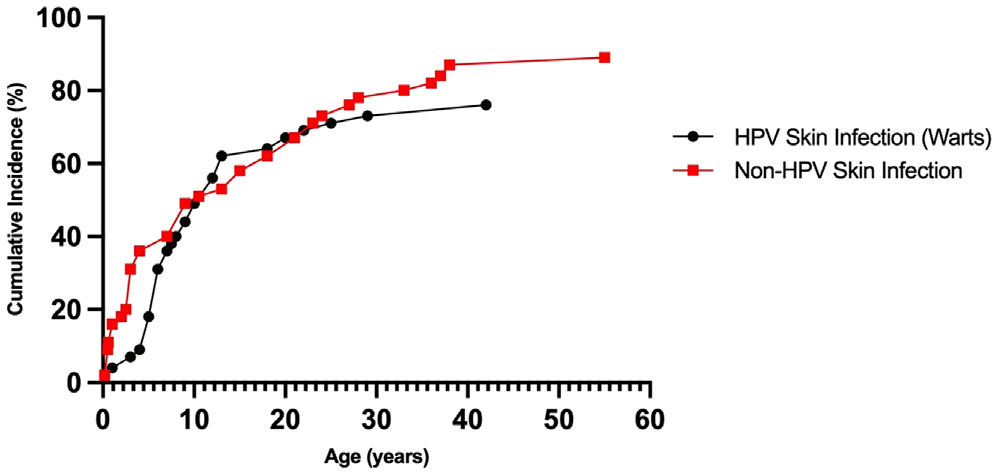
Cumulative incidence of first reported non‐human papillomavirus (HPV) and HPV skin infection. Age at reported onset for first documented non‐HPV and HPV skin infections were collected to calculate the cumulative incidence. Forty individuals had non‐HPV skin infection (mean age at onset 13.4 years, median age 9.0 years). Thirty‐four individuals had HPV skin infection in the cohort (mean onset 10.7 years, median 7.8 years). Sixteen of 40 patients (40%) with non‐HPV skin infection and eight of 34 patients (24%) with HPV skin infection had a first onset at 5 years or under. Twenty‐two of 40 patients (55%) with non‐HPV skin infection and 20 of 34 patients (59%) with HPV skin infection had a first onset before the age of 10 years.

The cumulative incidence of first reported HPV skin infection (warts) and non‐HPV skin infection were calculated and compared (Figure 2). The mean age at first reported warts was 10.7 years (median 7.8 years). Eight of 34 patients (24%) with warts were ≤5 years old at the time of diagnosis. Mean age of first reported non‐HPV skin infection was 13.4 years (median 9.0 years). Among those with non‐HPV skin infection, 16 of 40 patients (40%) were ≤5 years old, 32 patients in the cohort with WHIM experienced bacterial skin infections with a total of 122 bacterial infections recorded. The most common bacterial infections recorded included 41 abscesses, 37 general skin/soft tissue bacterial skin infections, and 29 cases of cellulitis. Less commonly reported bacterial skin infections included paronychias (*n* = 5), folliculitis (*n* = 4), pyomyositis (*n* = 3), purpura fulminans (*n* = 2), and omphalitis (*n* = 1). The most common species observed in the bacterial skin infections included *Staphyloccocus aureus* (including methicillin‐susceptible *Staphylococcus aureus* [MSSA] and methicillin‐resistant S*taphylococcus aureus* [MRSA]) and *Pseudomonas*. Other species recorded included *Pantoea* species, *Acinetobacter baumanii*, *Bartonella* spp, *Proteus mirabilis*, and *Streptococccus dysgalactiae* (Group G). Seven patients (16%) were diagnosed with skin cancer, including four (9%) with basal cell carcinoma (BCC), three (7%) with squamous cell carcinoma (SCC): two (4%) additional with SCC in situ of the genitals, and one (2%) with melanoma (Figure 1). The mean age among of the seven patients at their first reported skin cancer was 39 years (range, 28–58 years). A total of 41 skin cancers (32 BCCs, eight SCCs, and one melanoma) were observed among the seven individuals with skin cancer, with a mean of 5.9 skin cancers per patient (range, 1–22).

Chronic and inflammatory skin conditions were observed including psoriasis in four (9%), seborrheic dermatitis in six (13%), contact dermatitis in five (11%), nummular eczema/dermatitis in three (7%), and erythema nodosum in one (2%) patient. Thirteen patients (29%) had some form of other eczematous dermatitis and two (4%) were diagnosed with an idiopathic malar rash (Figure 1). These conditions were higher than expected for the size and age of the cohort.

### 3.2 Impact of CXCR4 pathogenic variant on cutaneous manifestations of WHIM syndrome

In this cohort, 12 distinct CXCR4 genetic variants were identified, including seven frameshift mutations, four truncation mutations, and one missense mutation. The most common variant in WHIM syndrome is the truncation mutation R334X, observed in 10 families in the cohort (Table 1). All seven individuals who developed skin cancer had truncation mutations: R334X in six patients and S338X in one patient. In the family with the missense mutation E343K, three patients did not have warts. Two of the three patients lacked other cutaneous manifestations. The remaining family members only had 1–2 cutaneous manifestations.

## 4 DISCUSSION

In the NIH WHIM cohort, a variety of skin infections, skin cancers, chronic skin conditions, and inflammatory skin disorders were observed. Both HPV infection, a hallmark of WHIM syndrome, and non‐HPV skin infections frequently manifest in early childhood. Specifically, 20 of 34 patients (59%) with HPV skin infection and 22 of 40 patients (55%) with non‐HPV skin infections developed symptoms before age 10. This early onset for both HPV and non‐HPV skin infection highlights their significance in the clinical presentation of WHIM syndrome. While HPV skin infection (warts) is an eponymous feature of WHIM, it is not fully penetrant, and non‐HPV skin infections may be as common and may occur at a similar early age.

A variety of bacterial, fungal, and viral skin infections were observed in patients with WHIM syndrome, raising the question of whether they are unable to mount a complete immunological response to infectious agents, thereby necessitating an immunological analysis. The recorded bacterial infections were numerous and likely to be an underrepresentation of the bacterial skin infections experienced. Additionally, one possible case of immunodeficiency‐related vaccine‐derived rubella virus with cutaneous granulomatous disease was recorded.

The most common variant, R334X, was associated with the most skin manifestations and the point mutation was associated with the fewest (Table S1). These findings may suggest that the CXCR4 variant type plays a role in determining the extent and severity of skin disease, but larger studies are needed to confirm this hypothesis.

Individuals with WHIM syndrome present with a broad spectrum of skin diseases, including skin infections, skin cancers, and inflammatory and chronic skin conditions. HPV and non‐HPV skin infections had a comparable early onset in our cohort. We hypothesize that CXCR4 variants may influence the susceptibility to, likelihood, and severity of skin diseases, but larger studies for association are needed. A comprehensive understanding of the myriad dermatological presentations of WHIM syndrome may facilitate early diagnosis and personalized treatment approaches to effectively manage the immunological underpinnings of the disease.

## CONFLICT OF INTEREST STATEMENT

None declared.

## ETHICS STATEMENT

An institutional reviewer board of the National Institutes of Health approved the study.

All patients provided informed consent prior to participation.

## TRIAL REGISTRATION

The present trial is registered under number NCT00128973.

## Supporting information


Data S1.

## Data Availability

The data that support the findings of this study are available from the corresponding author upon reasonable request.
