# Supplementary material for: Cutaneous manifestations of WHIM syndrome
Source: J Dermatol. 2025 Apr 9;52(5):917–21. doi: 10.1111/1346-8138.17733 (PMC12056281; doi:10.1111/1346-8138.17733)
Supplement: Supplementary file 1 — Data S1. [file JDE-52-917-s001.docx]

**Supplemental 1:**

**eTable 1.** Demographics of NIH WHIM Cohort

**eTable 2.** Location of Warts in NIH WHIM Cohort

**eTable 3.** Viral Skin Infection Observed in the NIH WHIM Cohort

**eTable 4.** Cutaneous Manifestations Observed in the NIH WHIM Cohort

**eFigure 1.** Photographs of skin manifestations in NIH WHIM Cohort

---

**eTable 1.** Demographics of NIH WHIM Cohort

New eTable 1: Demographics of NIH WHIM Cohort

| Subject | Status | Sex | Race/  Ethnicity | Age Diagnosis (years) | Current Age | CXCR4 Variant | Familial or DeNovo | Skin  Manifestations* |
| --- | --- | --- | --- | --- | --- | --- | --- | --- |
| 1 | Alive | F | White | 19.5 | 60 | R334X | Familial | 1,4,5,6 |
| 2 | Alive | F | White | 8.5 | 30 | R334X | Familial | 1,3,4,5 |
| 3 | Alive | F | White | 29.5 | 44 | R334X | Familial | 1,3,4,5,6 |
| 4 | Alive | F | Multiple Race | 10 | 24 | R334X | Familial | 1,4,5 |
| 5 | Alive | M | White | 4 | 19 | R334X | Familial | 1,2,3,4,5 |
| 6 | Alive | M | White | 48.5 | 65 | R334X | DeNovo | 1,2,4,5,6 |
| 7 | Alive | F | White | 28 | 42 | S324Vfs*20 | Familial | 1,2,3 |
| 8 | Alive | F | White | 0 | 15 | S324Vfs*20 | Familial | 2,3,4,7 |
| 9 | Deceased | F | White | 59 | 69 (age of death) | R334X | DeNovo | 1,2,5 |
| 10 | Alive | F | White | 48 | 34 | R334X | Familial | 1,3 |
| 11 | Alive | F | White | 24 | 36 | R334X | Familial | 1,2 |
| 12 | Alive | M | White | 2 | 16 | L329Qfs*13 | De Novo | 1,2,3,4 |
| 13 | Alive | F | White | 32 | 47 | E343K | Familial | 2 |
| 14 | Alive | M | White | 8 | 22 | E343K | Familial | 1 |
| 15 | Alive | F | White | 3 | 18 | E343K | Familial | 1,4 |
| 16 | Alive | M | White | 0.5 | 13 | E343K | Familial | None |
| 17 | Alive | F | White | 51 | 66 | E343K | De Novo | None |
| 18 | Alive | M | White | 44 | 66 | R334X | Familial | 1,3,4,6 |
| 19 | Alive | F | White | 18 | 31 | R334X | Familial | 1,3 |
| 20 | Alive | M | White | 4 | 30 | S324Vfs*20 | De Novo | 2,3,4 |
| 21 | Alive | F | Hispanic | 17 | 45 | S338X | De Novo | 2 |
| 22 | Alive | M | Hispanic | 1 | 22 | S338X | Familial | 1,2,3,4,5 |
| 23 | Alive | F | White | 41 | 62 | R334X | Familial | 1,2,3,5,6 |
| 24 | Alive | M | White | 6 | 27 | R334X | Familial | 1,2,3,4 |
| 25 | Alive | F | Black | 7 | 19 | G336X | De Novo | 3,4,5 |
| 26 | Alive | F | White | 4 | 15 | R334X | Familial | 1,2,3,4,5 |
| 27 | Deceased | M | White | 35 | 48 (age of death) | R334X | Familial | 1,3,5,6 |
| 28 | Alive | M | Hispanic | 14 | 25 | R334X | De Novo | 1,2,3,4,5 |
| 29 | Alive | M | Hispanic | 23 | 41 | R334X | De Novo | 1,2,3,4,5 |
| 30 | Alive | F | Hispanic | 0.5 | 16 | R334X | Familial | 1,3,5 |
| 31 | Alive | M | Multiple Race | 6 | 15 | K327Rfs*17 | Familial | 1,3 |
| 32 | Alive | M | Black | 28 | 36 | K327Rfs*17 | De Novo | 1,3,4,5 |
| 33 | Alive | F | White | 23 | 34 | E343X | Familial | 1,2,3,4,5 |
| 34 | Deceased | F | White | 54 | 61 (age of death) | E343X | Familial | 1,2,3,4,5 |
| 35 | Alive | F | White | 24 | 45 | S339Cfs*4 | De Novo | 1,3 |
| 36 | Alive | F | White | 0 | 15 | S339Cfs*4 | Familial | 7 |
| 37 | Alive | F | White | 36 | 45 | R334X | De Novo | 1,2,3,4,5 |
| 39 | Alive | F | White | 9 | 17 | R334X | Familial | 2,4 |
| 41 | Alive | F | White | 37 | 45 | V320Efs*23 | Familial | 1,2,3,5 |
| 42 | Alive | F | White | 14 | 22 | V320Efs*23 | Familial | 1,2,3,4 |
| 43 | Alive | M | White | 0 | 7 | R334X | Familial | 2,5 |
| 44 | Alive | M | White | 7 | 12 | S330Qfs*13 | De Novo | 1,2,3 |
| 45 | Alive | F | White | 37 | 43 | S338X | De Novo | 1,2,3,4,5,6 |
| 46 | Alive | M | White | 42 | 47 | R334X | De Novo | 1,2,3,4,5 |
| 47 | Alive | F | White | 7 | 11 | R332Qfs*22 | De Novo | 2,3,4 |
| Total: 45 | Alive: 42  Deceased:3 | F: 28  M: 17 | B:2  H:2  MR:2  W:36 | Avg: 20.4 years | Avg: 33.3 years [0-69] |  | Familial:29  De Novo: 16 |  |

Race and Ethnicity: Black (B), Hispanic (H), Multiple Race (MR), White (W); *Skin manifestations: 1 Warts, 2 Viral Skin Infections, 3 Bacterial Skin Infections, 4 Fungal Skin Infections, 5 Inflammatory Skin Findings, 6 Skin Cancer, 7 Bone Marrow Transplant

**eTable 2.** Location of Warts in NIH WHIM Cohort

| Location of Warts (n=34) | Patients affected: |
| --- | --- |
| Hands  (includes fingers, wrists) | 27 (60%) |
| Feet  (includes toes, ankles) | 20 (44%) |
| Genital/perianal/anogenital | 14 (31%) |
| Face & Scalp  ( lips, forehead, cheek, nose, frontal scalp) | 9 (20%) |
| Knees | 8 (18%) |
| Arms  (includes elbows) | 6 (13%) |
| Legs | 6 (13%) |
| Torso, Chest | 2 (4%) |
| Back, buttocks | 1 (2%) |

**eTable 3.** Viral Skin Infection Observed in the NIH WHIM Cohort

| Viral Skin Infections: | Patients affected: |
| --- | --- |
| Herpes Simplex Virus (HSV) | 11 (24%) |
| Molluscum Contagiosum | 9 (20%) |
| Varicella-Zoster Virus (VZV) | 8 (18%) |
| Hand Foot and Mouth Disease (Coxsackie) | 2 (4%) |
| Roseola (6th disease) | 2 (4%) |
| Rubella | 1 (2%) |

**eTable 4.** Cutaneous Manifestations Observed in the NIH WHIM Cohort

| **Cutaneous Manifestations Observed in the NIH WHIM Cohort** | N=45 |
| --- | --- |
| **Cutaneous Manifestation** | **Number of Patients affected, %** |
| **Skin Infections** |  |
| Warts (HPV) | 34 (76%) |
| Bacterial Skin Infection | 32 (71%) |
| Viral Skin Infection | 27 (60%) |
| Fungal Skin Infection | 25 (56%) |
| **Skin Cancer** |  |
| BCC | 4 (9%) |
| SCC/SCCis | 5 (11%) |
| Melanoma | 1 (2%) |
| **Chronic Inflammatory Skin Conditions** |  |
| Psoriasis | 4 (9%) |
| Alopecia  Traction alopecia  Androgenic alopecia with telogen effluvium  Alopecia with tinea corporis/capitis | 3 (7%)  1 (2%)  1 (2%)  1 (2%) |
| Malar rash, idiopathic | 2 (4%) |
| **Inflammatory Skin Findings** |  |
| Seborrheic dermatitis | 6 (13%) |
| Contact dermatitis (irritant or allergic) | 5 (11%) |
| Nummular eczema/dermatitis | 3 (7%) |
| Other eczematous dermatitis | 13 (29%) |
| Erythema nodosum | 1 (2%) |
| **Other Skin Findings** |  |
| Xerosis | 22 (49%) |
| Keratosis pilaris | 9 (20%) |
| Seborrheic keratosis | 6 (13%) |
| Hyperlinear palms | 4 (9%) |
| Café au lait macules | 4 (9%) |
| Dermatofibroma | 4 (9%) |
| Hemangioma/Cherry angioma | 3 (7%) |
|  |  |
|  |  |

**Supplemental 1 Tables Titles and Legends:**

**eTable 1.** Demographics of NIH WHIM Cohort

Demographic information for the cohort of 45 patients with WHIM Syndrome at the National Institutes of Health (NIH).

**eTable 2.** Location of Warts in NIH WHIM Cohort

Location of HPV skin infection (warts) in 34 of 45 patients with WHIM Syndrome in the National Institutes of Health (NIH) cohort, listed from most frequent to least frequent locations.

**eTable 3.** Viral Skin Infection Observed in the NIH WHIM Cohort

Types and frequencies of non-HPV viral skin infections (n=27) observed in the National Institutes of Health (NIH) cohort with WHIM Syndrome, including types and frequencies.

**eTable 4.** Cutaneous Manifestations Observed in the NIH WHIM Cohort

Cutaneous manifestations observed in 45 patients with WHIM Syndrome at the National Institutes of Health (NIH), categorized into 5 groups: skin infection, skin cancer, chronic inflammatory skin conditions, inflammatory skin findings, and other skin findings. For each category, the number and percentage of patients affected are displayed.

**Supplemental Figure 1.**

**1A**
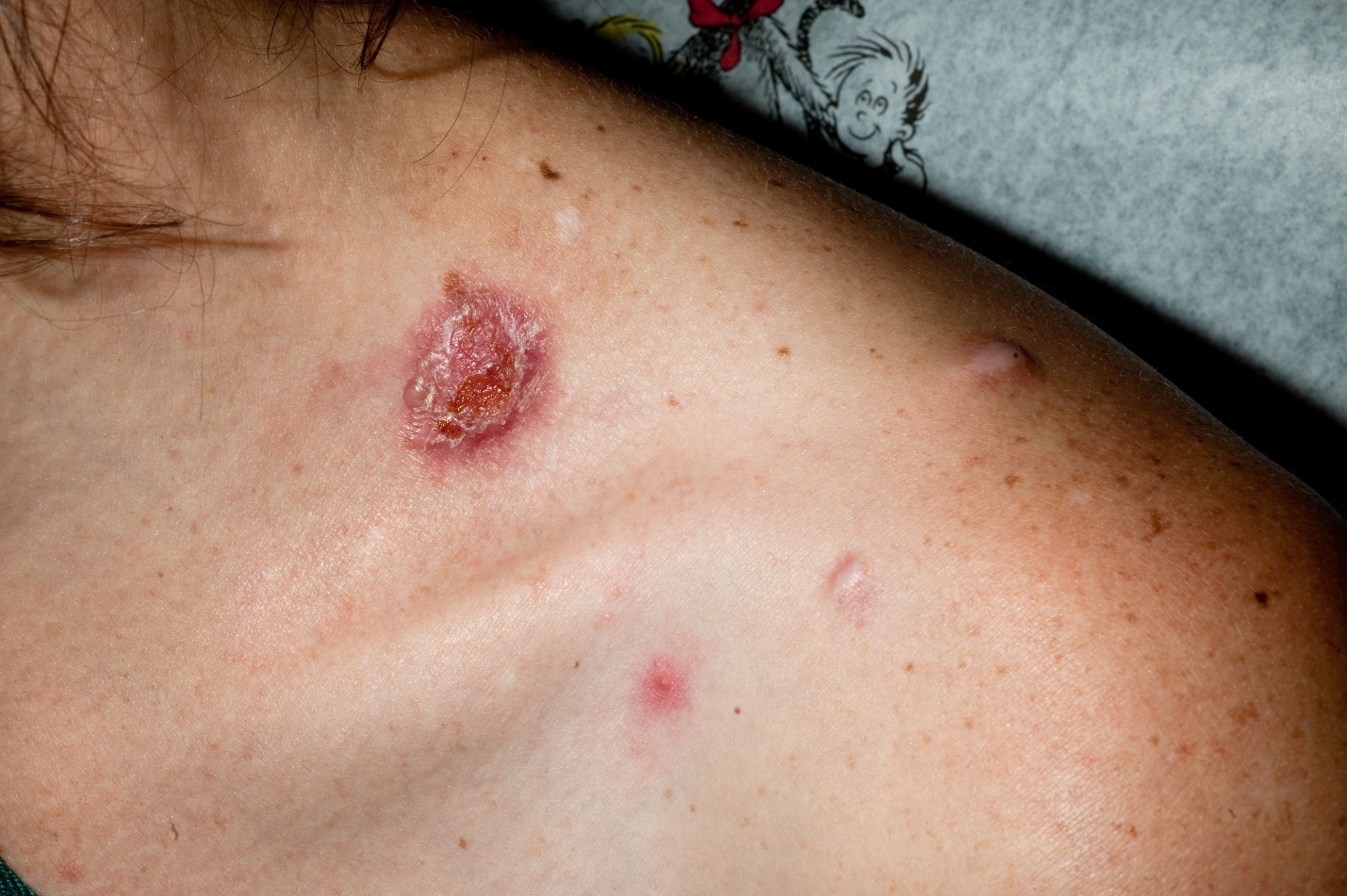


**1B**


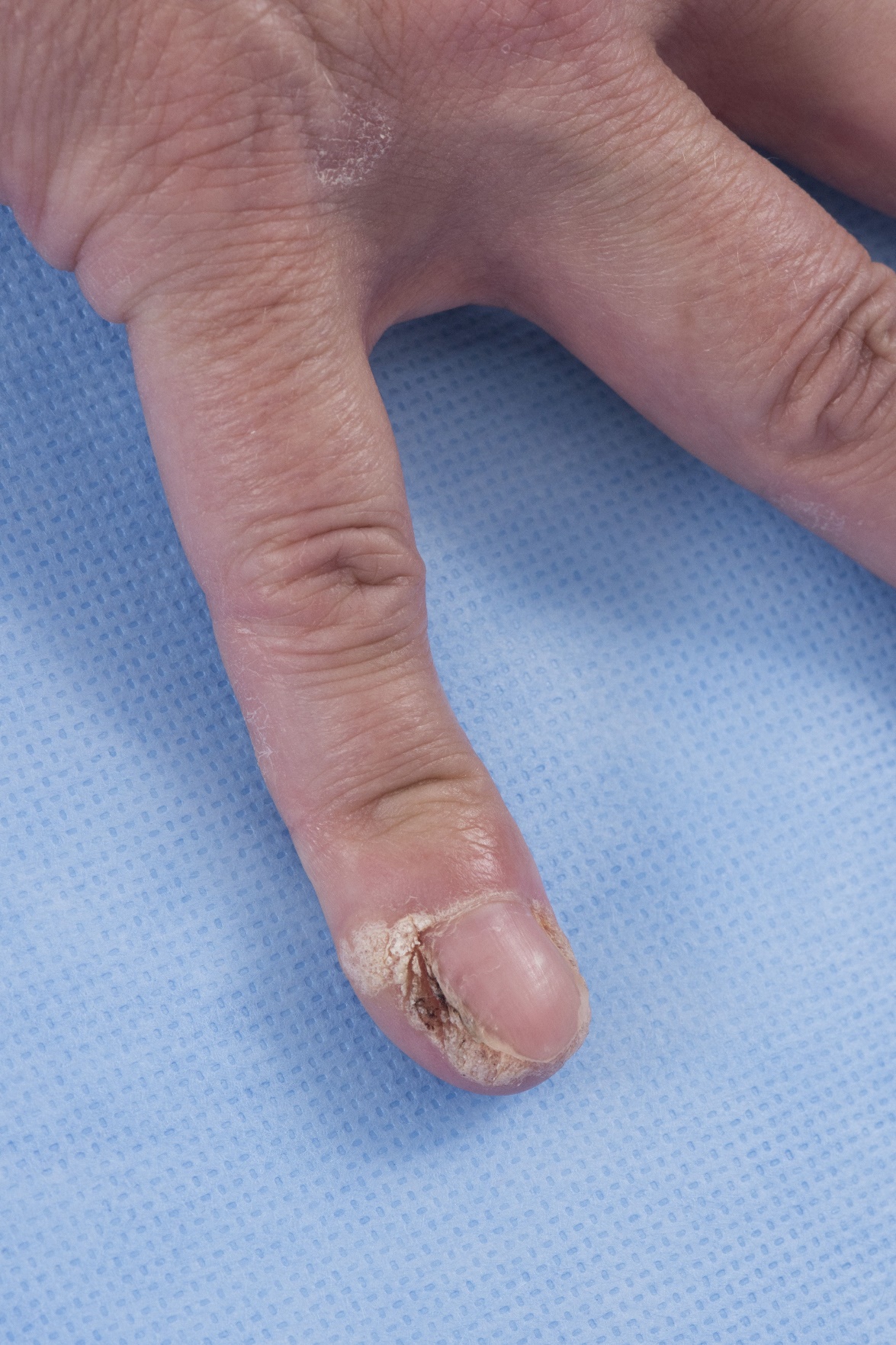


**1C**


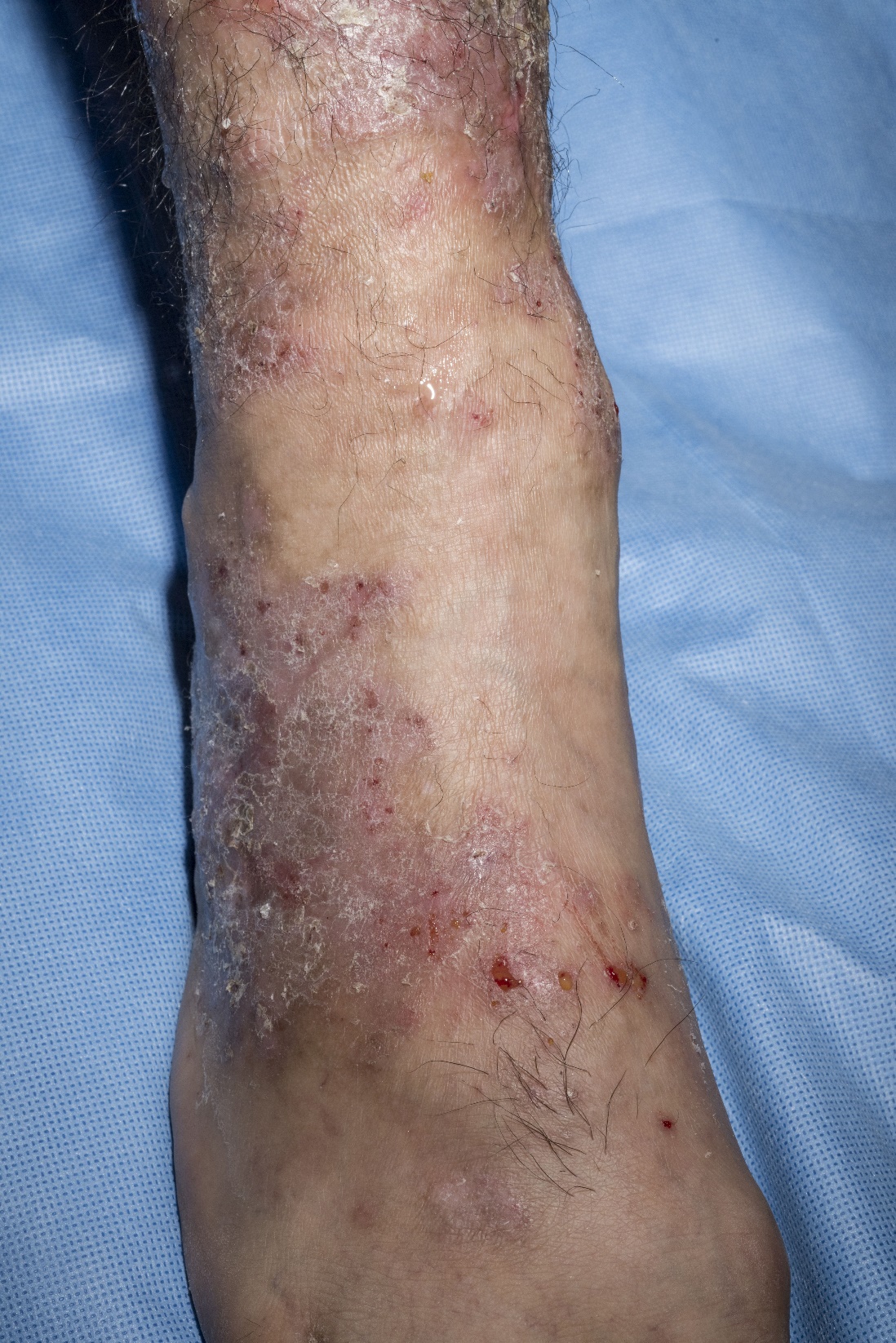


**1D**


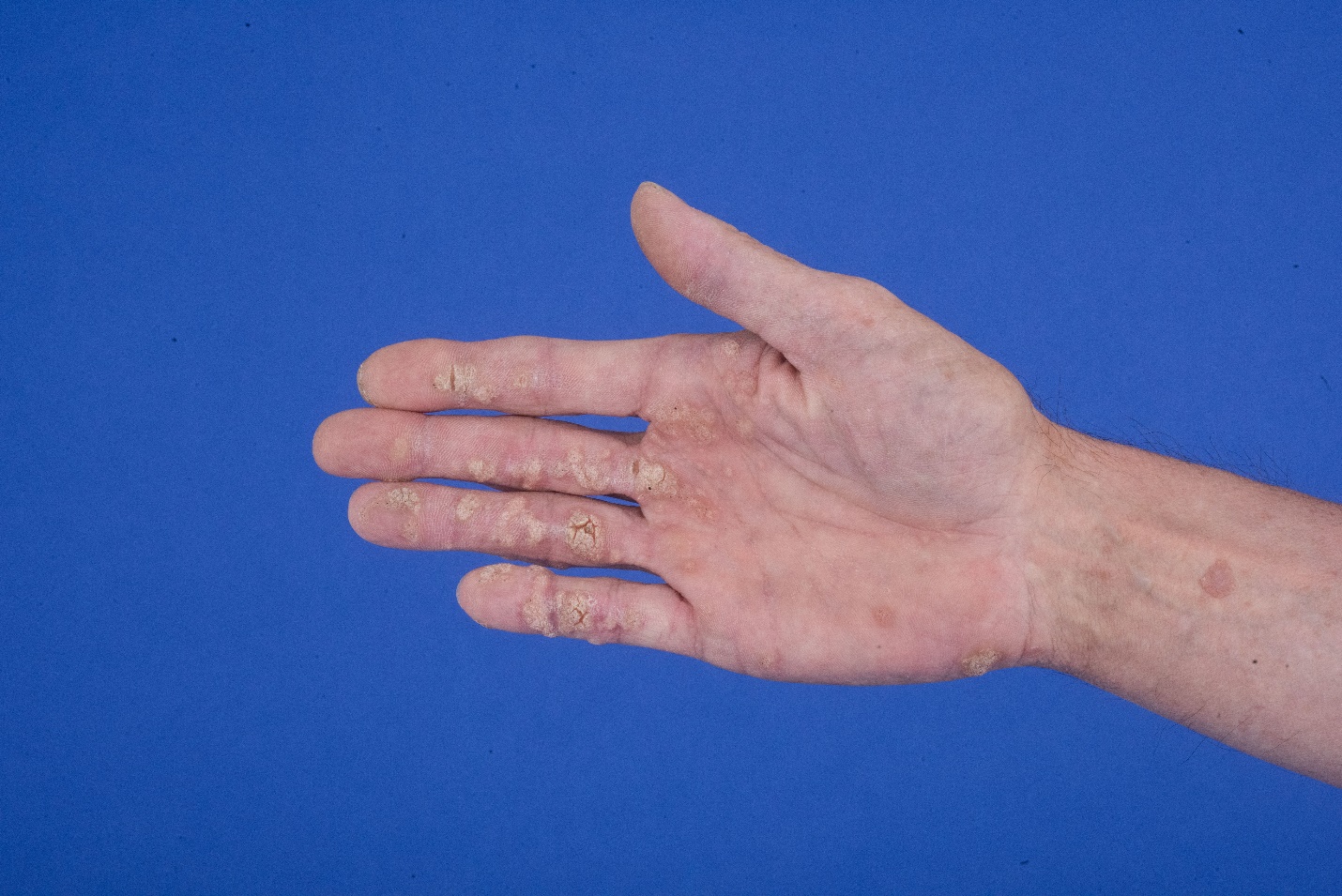


**1E**


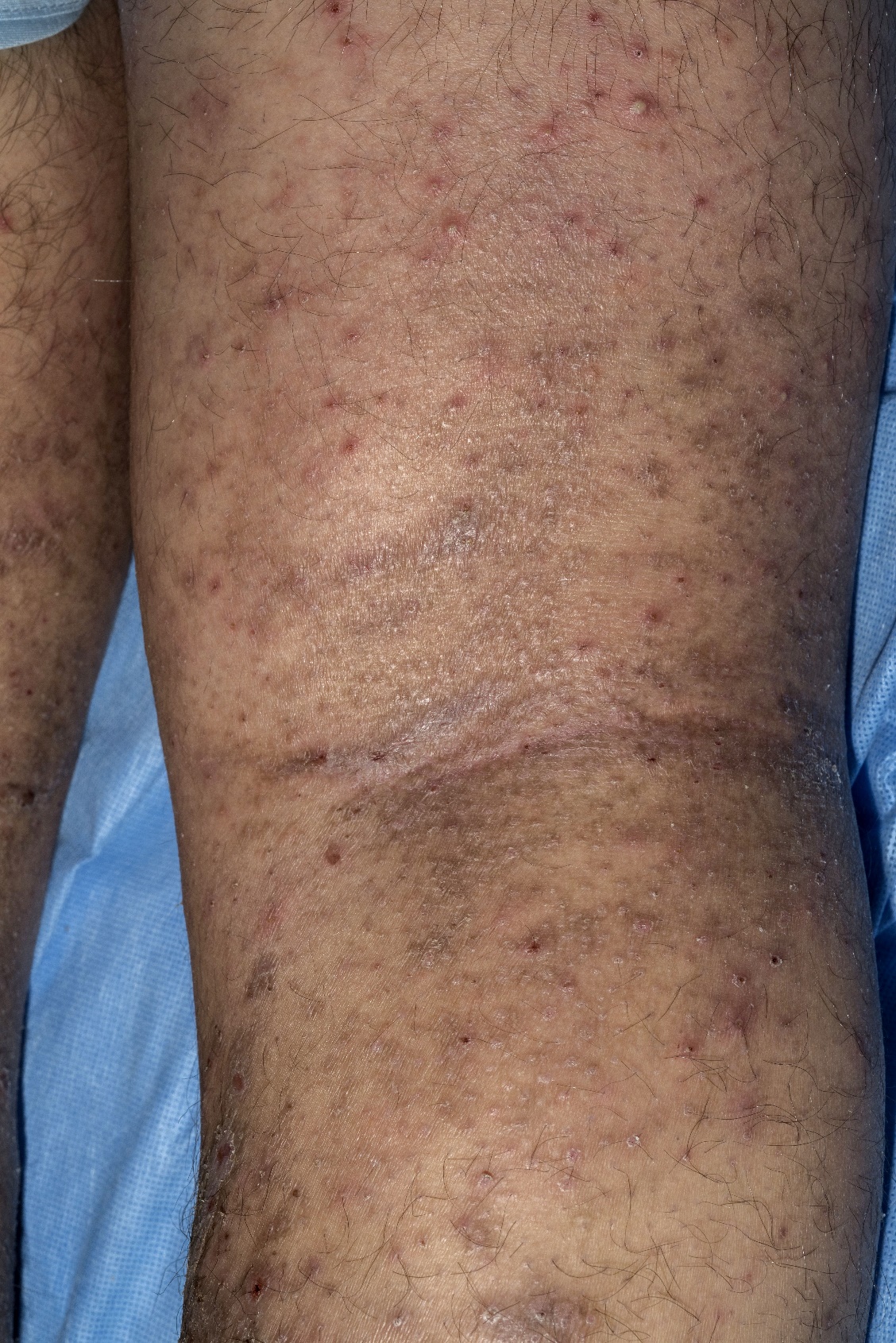


**eFigure1.** Photographs depicting skin changes noted in subjects with WHIM syndrome. **A.** Nodular basal cell carcinoma on the shoulder in subject age 30. **B.** Subungual wart on the digit. **C.** Eczematous dermatitis on the foot **D.** Multiple warts on the dorsal hand **E.** Folliculitis on the posterior leg.
